# Lamellar crystal structure of the trigonal mixed-metal phosphate RbCoFe(PO_4_)_2_

**DOI:** 10.1107/S2056989026007632

**Published:** 2026-10-02

**Authors:** Adam Bouraima, Said Ouaatta, Jamal Khmiyas, Jean jacques Anguilè, Thomas Makani, Abderrazzak Assani, Mohamed Saadi, Lahcen El Ammari

**Affiliations:** a Laboratoire de Chimie Appliquée des Matériaux Centre des Sciences des Matériaux Faculty of Science Mohammed V University in Rabat Avenue Ibn Batouta BP 1014 Rabat Morocco; b Laboratoire de Chimie des Matériaux Inorganiques Faculté des Sciences Département de Chimie Université des Sciences et Techniques de Masuku, BP 943 Franceville Gabon; Universidad de la República, Uruguay

**Keywords:** phosphate, sheet, crystal structure, X-ray diffraction

## Abstract

The title compound, rubidium cobalt iron bis­(phosphate), was synthesized by the solid-state method under atmospheric conditions. This phosphate crystallizes with trigonal symmetry in space group *P*3*m*1 and all atoms lie on special crystallographic positions. In this structure, the Co^II^O_4_ tetra­hedra alternate with one of the two types of PO_4_ tetra­hedra, sharing the vertices of their bases and extending along the *a*- and *b*-axis directions to form a first tetra­hedral layer, lying parallel to the (001) plane. The same alternation is observed between the Fe^III^O_4_ tetra­hedra and the other PO_4_ tetra­hedra, leading to the formation of a second layer also lying parallel to the (001) plane. In the first layer, all tetra­hedra are oriented downwards, while in the second, they are oriented upwards with respect to the *c* axis. The inter­connection between these two layers is ensured by the sharing of the vertices of the CoO_4_ and PO_4_ tetra­hedra, as well as the vertex of the FeO_4_ tetra­hedra, with those of the other PO_4_ tetra­hedra. The stacking of these two layers leads to the formation of [CoFeP_2_O_8_]^−^_∞_ double layers lying parallel to the (001) plane and creates hexa­gonal tunnels running parallel to the *c*-axis direction. The Rb^+^ cations are located at the inter­section of the tunnels with the inter­layer space, thus forming a plane lying perpendicular to the *c*-axis direction, sandwiched between the double layers. In this structure, each rubidium atom exhibits a coordination number of twelve, whereas in the similar structure of KMgFe(PO_4_)_2_, which has monoclinic symmetry, the potassium cation is surrounded by eight oxygen atoms.

## Abstract 2

In the title compound, all the cations occupy tetra­hedral sites, with the exception of rubidium, whose coordination number is twelve. The three types of tetra­hedra are linked at their vertices to form double layers stacked along the *c*-axis direction. The planes occupied by the rubidium ions are sandwiched between these layers.

## 1. Chemical context

In solid-state chemistry and materials science, transition-metal (TM) phosphates have been studied extensively, particularly those with open structures. These porous materials are inter­esting due to their potential industrial and environmental applications, ranging from catalysis to ion exchange and separation (Gier & Stucky, 1991 ▸; Maspoch *et al.*, 2007 ▸; Lin *et al.*, 2004 ▸), electrochemistry (Trad *et al.*, 2010 ▸) and magnetism (Ofer *et al.*, 2012 ▸; Hadouchi *et al.*, 2019 ▸). Among these, iron phosphates are particularly attractive due to their rich crystal chemistry (Moore, 1970 ▸; Redrup & Weller, 2009 ▸; Lajmi *et al.*, 2009 ▸) and variable physical properties (Elbouaanani *et al.*, 2002 ▸; Riou-Cavellec *et al.*, 1999 ▸). The structural diversity is mainly due to the ability of TM cations to adopt different oxidation states such as PbMn^II^_2_Mn^III^(PO_4_)_3_ (Alhakmi *et al.*, 2013 ▸) and coordination geometries, leading to a wide variety of families such as NaZnAl(PO_4_)_2_ (Yakubovich, *et al.*, 2019 ▸) and NaCuIn(PO_4_)_2_ (Benhsina *et al.*, 2020 ▸).

**Figure 1:**
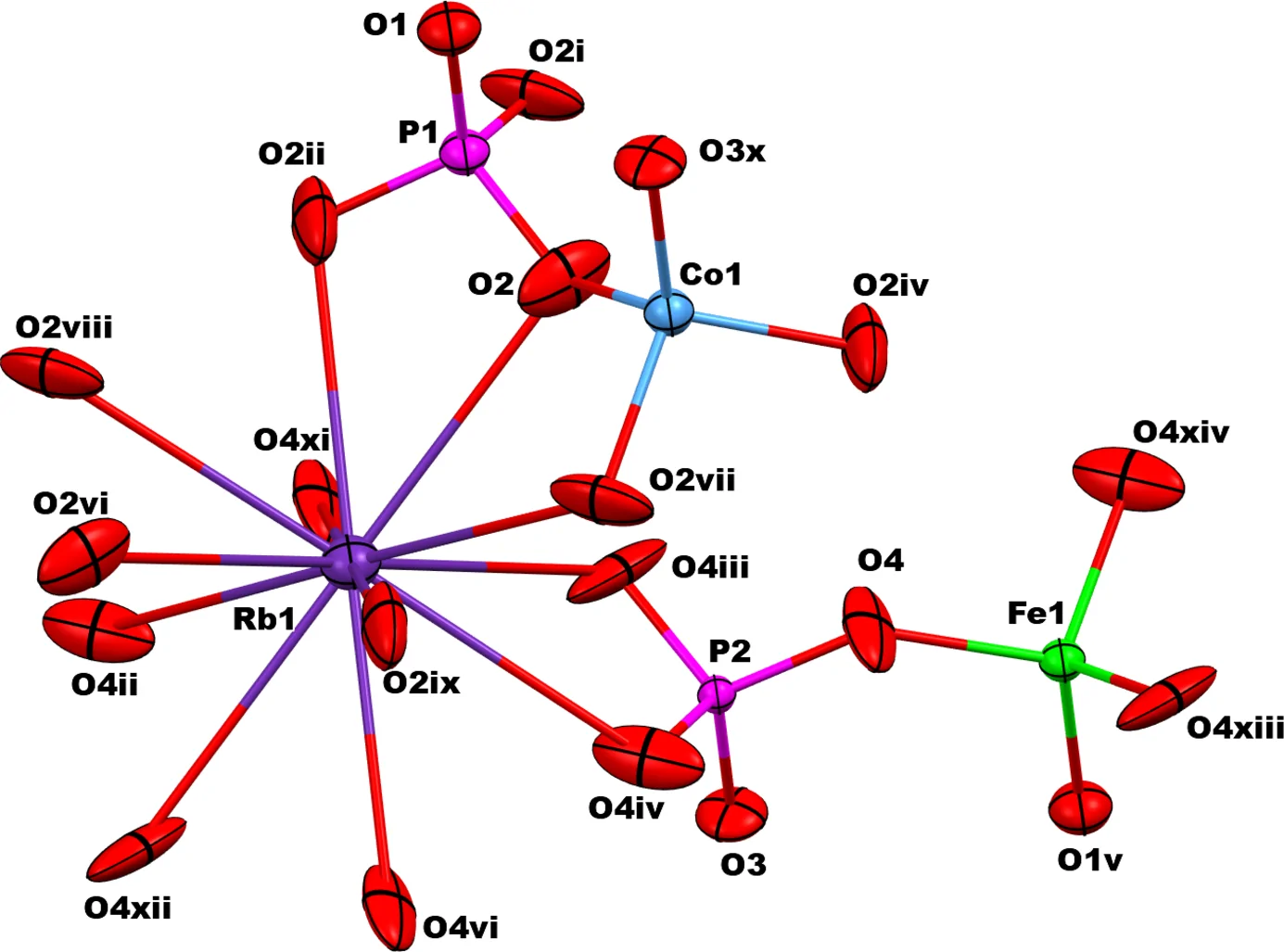
The principal polyhedra constituting the crystal structure of the compound RbCoFe(PO_4_)_2_, with atomic labels. Displacement ellipsoids are represented at the 50% probability level. Symmetry codes:(i) −*y* + 2, *x* − *y* + 1, *z*; (ii) −*x* + *y* + 1, −*x* + 2, *z*; (iii) −*y* + 1, *x* − *y*, *z*; (iv) −*x* + *y* + 1, −*x* + 1, *z*; (v) *x*, *y* − 1, *z* − 1; (vi) *x* − 1, *y*, *z*; (vii) −*y* + 1, *x* − *y*, *z*; (viii) −*y* + 1, *x* − *y* + 1, *z*; (ix) −*x* + *y*, −*x* + 1, *z*; (*x*) *x*, *y*, *z* + 1; (xi) *x*, *y* + 1, *z*; (xii) −*y*, *x* − *y*, *z*; (xiii) −*y* + 1, *x* − *y* − 1, *z*; (xiv) −*x* + *y* + 2, −*x* + 1, *z*.

Our research team has previously developed and characterized several new phosphates with the general formula *AMM*′(PO_4_)_2_, where *A*, *M*, and *M*′ represent alkali or alkaline earth cations, divalent and trivalent metals, respectively. Most of the compounds were obtained by solid-state reactions and identified by X-ray diffraction. Investigations of these materials reveal the richness and diversity of their structures. The most frequent structural types adopted by these crystals are alluaudite, ABW-zeolite, α-CrPO_4_, and others. The following compounds represent an example of each structural type, respectively, Na_2_*M*_2_Fe(PO_4_)_3_ (*M* = Co, Ni) (Bouraima *et al.*, 2015 ▸); β-KCoFe(PO_4_)_2_ (Bouraima *et al.*, 2022 ▸); BaNi_2_Fe(PO_4_)_3_ (Ouaatta *et al.*, 2023 ▸) and K_3_Ni_6_Fe(PO_4_)_6_ (Ouaatta *et al.*, 2019 ▸). As part of our ongoing studies in this area, we now describe the synthesis and characterization of the title compound RbCoFe(PO_4_)_2_ (**I**) (Fig. 1 ▸).

## 2. Structural commentary

In this structure, all atoms occupy special positions of the *P*3*m*1 space group. The asymmetric unit of the new phase consists of one Rb atom in Wyckoff position 1*b* (3*m*), Co1, P2, and O3 atoms in position 1*c* (3*m*), Fe1, P1, and O1 atoms in position 1*a* (3*m*), while O2 and O4 occupy Wyckoff position 3*d* (*m*). However, refining the structure with cobalt in Wyckoff position (1*a*) and iron in (1*c*) leads to inconclusive results. Reversing the positions of Fe in (1*a*) and Co in (1*c*) leads to slightly better results: the Co—O distances (Table 1 ▸) are slightly longer than the Fe—O distances, and the bond-valence sums are very close to the expected values. It is also noted that freely refining the occupancies of the Co/Fe sites by a mixture of Co^2+^ and Fe^3+^ leads to unstable refinement, and the refinement diverges. Refining the statistical distribution of the two cations Co^2+^ and Fe^3+^ at the two Wyckoff sites (1*a*) and (1*c*) converged well, with a slight improvement in the values of *R* (0.030) and *Rw*2 (0.077), but with very high standard deviations in the Co/Fe—O bond lengths. The same results are obtained when refining the structure by fixing different occupancy rates of the two sites by Co^2+^ and Fe^3+^. In light of the preceding analyses, we can conclude that the best model is the one corresponding to Fe^3+^ at (1*a*) and Co^2+^ at (1*c*).

**Figure 2:**
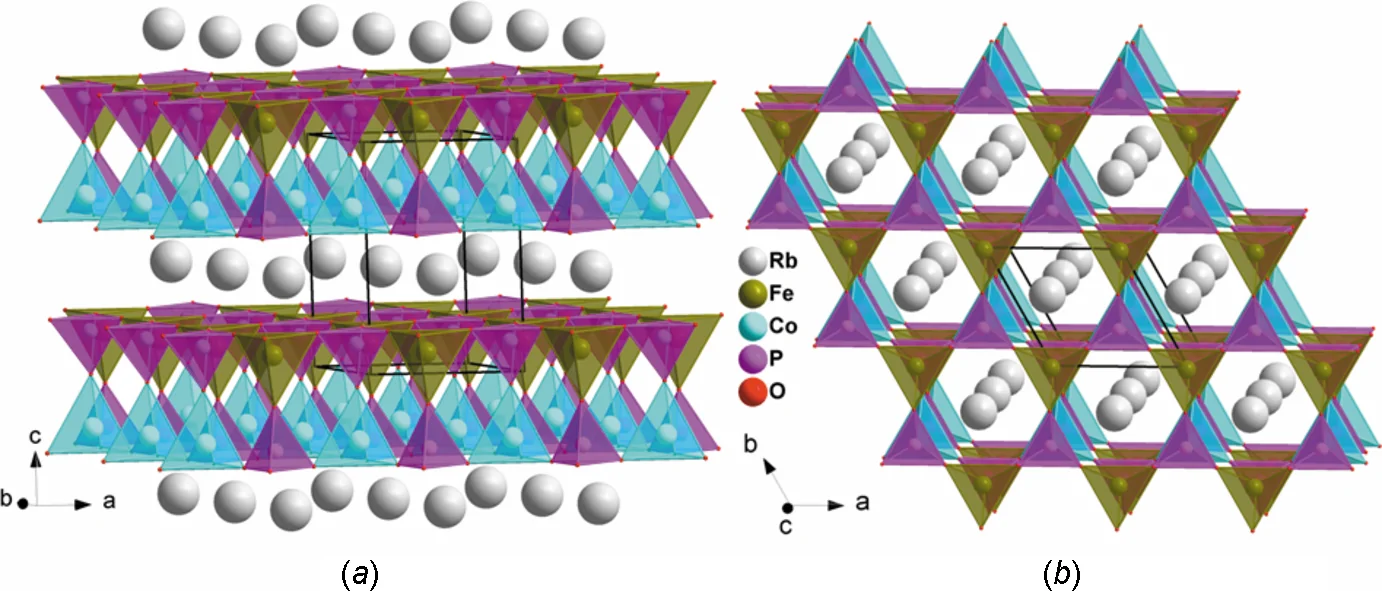
(*a*) Perspective view of the crystal structure of RbCoFe(PO_4_)_2_, showing the plane of Rb^+^ ions sandwiched between the double layers of tetra­hedra. (*b*) The Rb^+^ ions are located at the inter­section of the channels extending along the [001] direction with the inter­layer space.

Furthermore, all cations adopt a tetra­hedral environment, except for the rubidium ion, which is surrounded by twelve oxygen atoms. The average phospho­rus–oxygen bond lengths are 1.538 and 1.528 Å for P1 and P2, respectively. The average bond length for cobalt–oxygen is 1.887 Å, and 1.847 Å for the iron–oxygen distance. The crystal structure is built by two distinct types of sheets lying parallel to the *ab* plane. The CoO_4_ tetra­hedra alternate with P1O_4_ tetra­hedra, sharing vertices and extending along both the *a-* and *b-*axis directions to construct a first tetra­hedral sheet. All tetra­hedra within this layer are oriented upward relative to the *c* axis. These sheets exhibit hexa­gonal-shaped pores. The same alternation is observed between FeO_4_, and P2O_4_ tetra­hedra, leading to the formation of a second sheet, also encapsulating hexa­gonal-shaped pores. In this layer, the FeO_4_ and P2O_4_ tetra­hedra are oriented ‘down’ relative to the *c* axis. The inter­connection between these two sheets is ensured through vertex-sharing between CoO_4_ and P2O_4_, and FeO_4_ and P1O_4_ tetra­hedra *via* oxygen atoms (these inter-sheet bonds are constrained to be linear by symmetry). This arrangement leads to a [CoFeP_2_O_8_]^−^_∞_ double sheet lying parallel to the *ab* plane. The Rb^+^ cations form a plane sandwiched between the double layers. Each rubidium cation is surrounded by twelve oxygen atoms at the inter­section of the tunnels with the inter­layer space (Fig. 2 ▸).

The reliability of the structural model was validated using bond-valence-sum (BVS; Altermatt & Brown, 1985 ▸) and charge-distribution (CHARDI; Nespolo, 2016 ▸) methods. The CHARDI and BVS computations were performed with the *CHARDI2015* (Nespolo & Guillot, 2016 ▸) and *EXPO2014* (Altomare *et al.*, 2013 ▸) programs, respectively. The resulting parameters derived from both methods are listed in Table 2 ▸. The computed values *V*(*i*) (valence) and *Q*(*i*) (cationic charge) reveal excellent agreement between these values and their corresponding weighted oxidation numbers *q*(*i*)·sof(*i*). In accordance with this charge-distribution scheme, the cationic charge ratio *q*(*i*)/*Q*(*i*) ≃ 1 for all species, confirming the accuracy of this structural determination (Nespolo *et al.*, 1999 ▸). This model is also corroborated by the low of the mean absolute percentage deviation (MAPD = 2.9%), which assesses the agreement between the calculated CHARDI charges *Q*(*i*) and the expected formal ones *q*(*i*) (Eon & Nespolo, 2015 ▸).

In the crystal structure of RbCoFe(PO_4_)_2_, the coordination numbers (CN) and effective coordination numbers (ECoN) found for all the cationic sites are entirely consistent with the expected environments. For all crystallographic sites, the CN and ECoN values closely match, indicating well-defined coordination polyhedra, with the exception of the Rb1 atom, which exhibits a very low distortion level. Indeed, the Rb1 site adopts a high coordination number (CN = 12) with an ECoN value of 11.99, reflecting an almost ideal twelvefold coordination environment. This result confirms the existence of weakly directional bonds typical of alkali metal cations, where slight variations in bond lengths have a negligible impact on effective coordination. Both transition-metal sites, Co1 and Fe1, exhibit the same CN and ECoN values (CN = ECoN = 4.00), a feature of regular metal–oxygen bond lengths and consequently well-defined tetra­hedral coordination geometries. Similarly, the P1 and P2 sites, each display CN = 4 and ECoN = 4.00, confirming the presence of highly regular PO_4_ tetra­hedra.

Comparison of BVS results with the nominal valences *V*(*i*) confirms that the BVS approach leads to the expected values for the different crystallographic sites in the crystal structure of RbCoFe(PO_4_)_2_. The global instability index (GII) was also calculated to assess the electrostatic plausibility and the overall stability of the crystal structure (Salinas-Sanchez *et al.*, 1992 ▸). The resulting GII value of 0.108 suggests a structurally stable and rigid framework, thereby confirming the reliability of the structural model.

The title compound crystallizes in a non-centrosymmetric space group with a polar axis (*P*3*m*1), which allows it to be classified among compounds likely to have ferroelectric properties (Abrahams, 2008 ▸). The ferroelectric paraelectric phase transition requires a space group change from *P*3*m*1 to one of its the non-isomorphic supergroups *P*
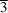
*m*1, *P*
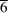
*m*2, *P*6_3_*mc*, or *P*6*mm*. Analysis of atomic positions using *PLATON* (Spek, 2020 ▸) shows that the structure could exhibit an approximate/pseudo-inversion symmetry (Marsh, 1994 ▸).

## 3. Database survey

A survey of the Powder Diffraction database (PDF2; version 2023) and of the Inorganic Crystal Structure Database (ICSD; version 5.5.0 from FIZ Karlsruhe) shows that among phosphates of the type *AMM*′(PO_4_)_2_ (*A* = K or Rb, *M* = divalent cation and *M*′ = trivalent cation), to our knowledge only RbCoFe(PO_4_)_2_ crystallizes in a trigonal symmetry (space group *P*3m1) with *a* = 5.4474 Å and *c* = 8.1547 Å.

A number of known phosphates show similar unit cells, such as: β-CaZn_2_(PO_4_)_2_ (*a* = 5.180 Å, *c* = 7.670 Å, space group *P*3; Kreidler & Hummel, 1967 ▸; Czaya, 1972 ▸), SrMg_2_(PO_4_)_2_ (*a* = 5.133 Å, *c* = 8.038 Å, space group *P*3*m*; CPDS N 52–1587), and BaMg_2_(PO_4_)_2_ (*a* = 5.236 Å, *c* = 8.201 Å, space group *P*3*m*; JCPDS N 52–1587), space group *P*3*m*1), β-NaZnPO_4_ (*a* = 5.101 Å, *c* = 8.365 Å, space group *P*6_3_; JCPDS N 52-0483) and NaBaPO_4_ (*a* = 5.622 Å, *c* = 7.259 Å, space group *P*3*m*1; Calvo & Faggiani, 1975 ▸; Launay *et al.*, 1992 ▸). Despite these similarities, the structures of these compounds differ from that studied here. In these materials, the polyhedra form rings around the large cations, whereas in the structure of RbCoFe(PO_4_)_2_, the tetra­hedra are ordered and form a double layer surrounding the rubidium plane. It may be noted that this lamellar structure is adopted by *AM*Fe(PO_4_)_2_ phosphates (*M* = Mg, Co, Zn and *A* = K or Rb), although the latter crystallize in the monoclinic system, space group *C*2/*c*.

## 4. Synthesis and crystallization

The title compound was synthesized by mixing cobalt nitrate [Co(NO_3_)·6H_2_O], iron nitrate [Fe(NO_3_)_3_·9H_2_O], ortho­phospho­ric acid (H_3_PO_4_) and rubidium carbonate (Rb_2_CO_3_) in the molar ratio 1:1:1:2. The mixture was placed in a small beaker containing distilled water, stirred for 24 h at room temperature, and subsequently evaporated to dryness. The resulting reaction mixture was heat-treated at 573 K and 773 K before being thoroughly ground in an agate mortar. The resulting solid was then transferred into a platinum crucible and heated to the melting temperature of 1233 K. After holding this temperature for 15 min, the molten product was cooled gradually to 1073 K at a rate of 5 K h^−1^, and then quickly to room temperature by shutting off the furnace. The final product comprised purple single crystals of suitable size for X-ray diffraction analysis, corresponding to the title phosphate.

## 5. Refinement

Crystal data, data collection and structure refinement details are given in Table 3 ▸. The crystal structure was refined as an inversion twin using the TWIN/BASF instructions in *SHELXL*. The refined Flack parameter is 0.50 (7), indicating a near-perfect two-component inversion twin. The largest residual electron-density peak and the deepest hole in the last difference-Fourier map are located 0.53 and 0.17 Å from atoms O2 and O4, respectively.

## Supplementary Material

Crystal structure: contains datablock(s) I. DOI: 10.1107/S2056989026007632/oo2018sup1.cif

Structure factors: contains datablock(s) I. DOI: 10.1107/S2056989026007632/oo2018Isup2.hkl

CCDC reference: 2576725

Additional supporting information:  crystallographic information; 3D view; checkCIF report

Additional supporting information:  crystallographic information; 3D view; checkCIF report

## Figures and Tables

**Table 1:** Selected geometric parameters (Å, °)

Rb1—O2	3.220 (5)	Co1—O2	1.893 (7)
Rb1—O4^i^	3.245 (5)	P1—O2	1.5373 (14)
Fe1—O1^ii^	1.844 (9)	P1—O1	1.5398 (15)
Fe1—O4	1.848 (6)	P2—O3	1.500 (9)
Co1—O3^iii^	1.870 (13)	P2—O4	1.5374 (14)
			
P1—O1—Fe1^iv^	180.0	P2—O3—Co1^v^	180.0
P1—O2—Co1	133.3 (6)	P2—O4—Fe1	137.0 (6)

Symmetry codes: (i) 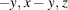; (ii) 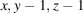; (iii) 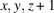; (iv) 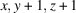; (v) 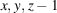.

**Table 2:** CHARDI and BVS results for the principal cation species in RbCoFe(PO_4_)_2_ *q*(*i*) = formal oxidation number; sof(*i*) = site occupancy; CN(*i*) = classical coordination number; *Q*(*i*) = calculated charge; *V*(*i*) = calculated valence; ECoN(*i*) = effective coordination number.

Cation	*q*(*i*)·sof(*i*)	CN(*i*)	ECoN(*i*)	*V*(*i*)	*Q*(*i*)	*q*(*i*)/*Q*(*i*)
Rb1	1	12	11.99	0.87	1.00	1.00
Co1	2	4	4.00	2.02	2.00	0.96
Fe1	3	4	4.00	3.17	2.88	1.04
P1	5	4	4.00	4.95	5.17	0.97
P2	5	4	3.98	5.09	4.87	1.03

**Table 3:** Experimental details

Crystal data	
Chemical formula	RbCoFe(PO_4_)_2_
*M*_r_	390.19
Crystal system, space group	Trigonal, *P*3*m*1
Temperature (K)	293
*a*, *c* (Å)	5.4474 (2), 8.1547 (3)
*V* (Å^3^)	209.56 (2)
*Z*	1
Radiation type	Mo *K*α
μ (mm^−1^)	9.87
Crystal size (mm)	0.25 × 0.21 × 0.14

Data collection	
Diffractometer	Bruker X8 *APEX* Diffractometer
Absorption correction	Multi-scan (*SADABS*; Krause *et al.*, 2015 ▸)
*T*_min_, *T*_max_	0.507, 0.748
No. of measured, independent and observed [*I* > 2σ(*I*)] reflections	14543, 945, 844
*R*_int_	0.039
(sin θ/λ)_max_ (Å^−1^)	0.862

Refinement	
*R*[*F*^2^ > 2σ(*F*^2^)], *wR*(*F*^2^), *S*	0.032, 0.079, 1.11
No. of reflections	945
No. of parameters	35
No. of restraints	4
Δρ_max_, Δρ_min_ (e Å^−3^)	1.30, −0.95
Absolute structure	Refined as an inversion twin
Absolute structure parameter	0.50 (7)

Computer programs: *APEX3* and *SAINT* (Bruker, 2016 ▸), *SHELXT2014/4* (Sheldrick, 2015*a* ▸), *SHELXL2019/3* (Sheldrick, 2015*b* ▸), *WinGX* (Farrugia, 2012 ▸), *DIAMOND* (Brandenburg, 2006 ▸) and *publCIF* (Westrip, 2010 ▸).

## supplementary crystallographic information

### Rubidium cobalt iron bis(phosphate) Crystal data

**Table:**

RbCoFe(PO_4_)_2_	*D*_x_ = 3.092 Mg m^−^^3^
*M**_r_* = 390.19	Mo *K*α radiation, λ = 0.71073 Å
Trigonal, *P*3*m*1	Cell parameters from 945 reflections
*a* = 5.4474 (2) Å	θ = 2.5–37.8°
*c* = 8.1547 (3) Å	µ = 9.87 mm^−^^1^
*V* = 209.56 (2) Å^3^	*T* = 293 K
*Z* = 1	Block, purple
*F*(000) = 184	0.25 × 0.21 × 0.14 mm

### Rubidium cobalt iron bis(phosphate) Data collection

**Table:**

Bruker X8 APEX Diffractometer	945 independent reflections
Radiation source: fine-focus sealed tube	844 reflections with *I* > 2σ(*I*)
Graphite monochromator	*R*_int_ = 0.039
φ and ω scans	θ_max_ = 37.8°, θ_min_ = 2.5°
Absorption correction: multi-scan (SADABS; Krause *et al.*, 2015)	*h* = −9→9
*T*_min_ = 0.507, *T*_max_ = 0.748	*k* = −9→9
14543 measured reflections	*l* = −14→14

### Rubidium cobalt iron bis(phosphate) Refinement

**Table:**

Refinement on *F*^2^	Primary atom site location: dual
Least-squares matrix: full	*w* = 1/[σ^2^(*F*_o_^2^) + (0.0284*P*)^2^ + 0.4865*P*] where *P* = (*F*_o_^2^ + 2*F*_c_^2^)/3
*R*[*F*^2^ > 2σ(*F*^2^)] = 0.032	(Δ/σ)_max_ < 0.001
*wR*(*F*^2^) = 0.079	Δρ_max_ = 1.30 e Å^−^^3^
*S* = 1.11	Δρ_min_ = −0.95 e Å^−^^3^
945 reflections	Absolute structure: Refined as an inversion twin
35 parameters	Absolute structure parameter: 0.50 (7)
4 restraints	

### Rubidium cobalt iron bis(phosphate) Special details

**Table:**

Geometry. All esds (except the esd in the dihedral angle between two l.s. planes) are estimated using the full covariance matrix. The cell esds are taken into account individually in the estimation of esds in distances, angles and torsion angles; correlations between esds in cell parameters are only used when they are defined by crystal symmetry. An approximate (isotropic) treatment of cell esds is used for estimating esds involving l.s. planes.
Refinement. Refined as a 2-component inversion twin.

### Rubidium cobalt iron bis(phosphate) Fractional atomic coordinates and isotropic or equivalent isotropic displacement parameters (Å^2^)

**Table:**

	*x*	*y*	*z*	*U*_iso_*/*U*_eq_	
Rb1	0.333333	0.666667	0.4375 (7)	0.02836 (19)	
Fe1	1.000000	0.000000	0.1327 (6)	0.0167 (6)	
Co1	0.666667	0.333333	0.7444 (6)	0.0204 (6)	
P1	1.000000	1.000000	0.7177 (5)	0.0198 (11)	
P2	0.666667	0.333333	0.1577 (4)	0.0127 (8)	
O1	1.000000	1.000000	0.9066 (5)	0.029 (5)	
O2	0.8489 (5)	0.6978 (9)	0.6473 (11)	0.052 (4)	
O3	0.666667	0.333333	−0.0263 (12)	0.033 (6)	
O4	0.8199 (4)	0.1801 (4)	0.2218 (10)	0.055 (4)	

### Rubidium cobalt iron bis(phosphate) Atomic displacement parameters (Å^2^)

**Table:**

	*U*^11^	*U*^22^	*U*^33^	*U*^12^	*U*^13^	*U*^23^
Rb1	0.0310 (3)	0.0310 (3)	0.0230 (3)	0.01552 (13)	0.000	0.000
Fe1	0.0175 (9)	0.0175 (9)	0.0153 (13)	0.0087 (4)	0.000	0.000
Co1	0.0207 (9)	0.0207 (9)	0.0198 (14)	0.0104 (4)	0.000	0.000
P1	0.0211 (15)	0.0211 (15)	0.017 (2)	0.0106 (7)	0.000	0.000
P2	0.0121 (11)	0.0121 (11)	0.0139 (18)	0.0061 (5)	0.000	0.000
O1	0.033 (7)	0.033 (7)	0.020 (7)	0.016 (4)	0.000	0.000
O2	0.083 (9)	0.018 (5)	0.035 (5)	0.009 (2)	0.0034 (18)	0.007 (4)
O3	0.044 (9)	0.044 (9)	0.011 (5)	0.022 (4)	0.000	0.000
O4	0.099 (10)	0.099 (10)	0.019 (3)	0.088 (10)	−0.0049 (17)	0.0049 (17)

### Rubidium cobalt iron bis(phosphate) Geometric parameters (Å, º)

**Table:**

Rb1—O2^i^	3.220 (5)	Fe1—O4^xi^	1.848 (6)
Rb1—O2^ii^	3.220 (5)	Fe1—O4	1.848 (6)
Rb1—O2^iii^	3.220 (5)	Co1—O3^xii^	1.870 (13)
Rb1—O2	3.220 (5)	Co1—O2^vi^	1.893 (7)
Rb1—O2^iv^	3.220 (5)	Co1—O2^v^	1.893 (7)
Rb1—O2^v^	3.220 (5)	Co1—O2	1.893 (7)
Rb1—O4^vi^	3.245 (5)	P1—O2	1.5373 (14)
Rb1—O4^v^	3.245 (5)	P1—O2^xiii^	1.5373 (15)
Rb1—O4^iii^	3.245 (5)	P1—O2^i^	1.5373 (14)
Rb1—O4^vii^	3.245 (5)	P1—O1	1.5398 (15)
Rb1—O4^viii^	3.245 (5)	P2—O3	1.500 (9)
Rb1—O4^i^	3.245 (5)	P2—O4	1.5374 (14)
Fe1—O1^ix^	1.844 (9)	P2—O4^v^	1.5374 (14)
Fe1—O4^x^	1.848 (6)	P2—O4^vi^	1.5374 (14)
			
O2^i^—Rb1—O2^ii^	55.1 (2)	O4^x^—Fe1—Rb1^xvi^	52.81 (12)
O2^i^—Rb1—O2^iii^	94.4 (2)	O4^xi^—Fe1—Rb1^xvi^	52.81 (12)
O2^ii^—Rb1—O2^iii^	45.10 (12)	O4—Fe1—Rb1^xvi^	118.5 (2)
O2^i^—Rb1—O2	45.10 (12)	Rb1^xiv^—Fe1—Rb1^xvi^	85.60 (7)
O2^ii^—Rb1—O2	94.4 (2)	Rb1^xv^—Fe1—Rb1^xvi^	85.60 (7)
O2^iii^—Rb1—O2	115.6 (3)	O3^xii^—Co1—O2^vi^	114.7 (2)
O2^i^—Rb1—O2^iv^	115.6 (3)	O3^xii^—Co1—O2^v^	114.7 (2)
O2^ii^—Rb1—O2^iv^	94.4 (2)	O2^vi^—Co1—O2^v^	103.7 (3)
O2^iii^—Rb1—O2^iv^	55.1 (2)	O3^xii^—Co1—O2	114.7 (2)
O2—Rb1—O2^iv^	94.4 (2)	O2^vi^—Co1—O2	103.7 (3)
O2^i^—Rb1—O2^v^	94.4 (2)	O2^v^—Co1—O2	103.7 (3)
O2^ii^—Rb1—O2^v^	115.6 (3)	O3^xii^—Co1—Rb1	128.51 (5)
O2^iii^—Rb1—O2^v^	94.4 (2)	O2^vi^—Co1—Rb1	116.8 (2)
O2—Rb1—O2^v^	55.1 (2)	O2^v^—Co1—Rb1	51.99 (12)
O2^iv^—Rb1—O2^v^	45.10 (12)	O2—Co1—Rb1	51.99 (12)
O2^i^—Rb1—O4^vi^	125.48 (7)	O3^xii^—Co1—Rb1^xv^	128.51 (5)
O2^ii^—Rb1—O4^vi^	179.2 (3)	O2^vi^—Co1—Rb1^xv^	51.99 (12)
O2^iii^—Rb1—O4^vi^	134.74 (4)	O2^v^—Co1—Rb1^xv^	51.99 (12)
O2—Rb1—O4^vi^	86.37 (11)	O2—Co1—Rb1^xv^	116.8 (2)
O2^iv^—Rb1—O4^vi^	85.85 (12)	Rb1—Co1—Rb1^xv^	85.33 (7)
O2^v^—Rb1—O4^vi^	65.15 (8)	O3^xii^—Co1—Rb1^xiv^	128.51 (5)
O2^i^—Rb1—O4^v^	85.85 (12)	O2^vi^—Co1—Rb1^xiv^	51.99 (12)
O2^ii^—Rb1—O4^v^	134.74 (4)	O2^v^—Co1—Rb1^xiv^	116.8 (2)
O2^iii^—Rb1—O4^v^	179.2 (3)	O2—Co1—Rb1^xiv^	51.99 (12)
O2—Rb1—O4^v^	65.15 (8)	Rb1—Co1—Rb1^xiv^	85.33 (7)
O2^iv^—Rb1—O4^v^	125.48 (7)	Rb1^xv^—Co1—Rb1^xiv^	85.33 (7)
O2^v^—Rb1—O4^v^	86.37 (11)	O2—P1—O2^xiii^	106.9 (5)
O4^vi^—Rb1—O4^v^	45.39 (10)	O2—P1—O2^i^	106.9 (5)
O2^i^—Rb1—O4^iii^	179.2 (3)	O2^xiii^—P1—O2^i^	106.9 (5)
O2^ii^—Rb1—O4^iii^	125.48 (7)	O2—P1—O1	111.9 (4)
O2^iii^—Rb1—O4^iii^	86.37 (11)	O2^xiii^—P1—O1	111.9 (4)
O2—Rb1—O4^iii^	134.74 (4)	O2^i^—P1—O1	111.9 (4)
O2^iv^—Rb1—O4^iii^	65.15 (8)	O2—P1—Rb1	53.5 (2)
O2^v^—Rb1—O4^iii^	85.85 (12)	O2^xiii^—P1—Rb1	122.1 (5)
O4^vi^—Rb1—O4^iii^	53.9 (2)	O2^i^—P1—Rb1	53.5 (2)
O4^v^—Rb1—O4^iii^	93.4 (2)	O1—P1—Rb1	126.00 (11)
O2^i^—Rb1—O4^vii^	65.15 (8)	O2—P1—Rb1^xvii^	122.1 (5)
O2^ii^—Rb1—O4^vii^	86.37 (11)	O2^xiii^—P1—Rb1^xvii^	53.5 (2)
O2^iii^—Rb1—O4^vii^	125.48 (7)	O2^i^—P1—Rb1^xvii^	53.5 (2)
O2—Rb1—O4^vii^	85.85 (12)	O1—P1—Rb1^xvii^	126.00 (11)
O2^iv^—Rb1—O4^vii^	179.2 (3)	Rb1—P1—Rb1^xvii^	88.95 (15)
O2^v^—Rb1—O4^vii^	134.74 (4)	O2—P1—Rb1^xiv^	53.5 (2)
O4^vi^—Rb1—O4^vii^	93.4 (2)	O2^xiii^—P1—Rb1^xiv^	53.5 (2)
O4^v^—Rb1—O4^vii^	53.9 (2)	O2^i^—P1—Rb1^xiv^	122.1 (5)
O4^iii^—Rb1—O4^vii^	114.1 (3)	O1—P1—Rb1^xiv^	126.00 (11)
O2^i^—Rb1—O4^viii^	134.74 (4)	Rb1—P1—Rb1^xiv^	88.95 (15)
O2^ii^—Rb1—O4^viii^	85.85 (12)	Rb1^xvii^—P1—Rb1^xiv^	88.95 (15)
O2^iii^—Rb1—O4^viii^	65.15 (8)	O3—P2—O4	109.9 (4)
O2—Rb1—O4^viii^	179.2 (3)	O3—P2—O4^v^	109.9 (4)
O2^iv^—Rb1—O4^viii^	86.37 (11)	O4—P2—O4^v^	109.1 (4)
O2^v^—Rb1—O4^viii^	125.48 (7)	O3—P2—O4^vi^	109.9 (4)
O4^vi^—Rb1—O4^viii^	93.4 (2)	O4—P2—O4^vi^	109.1 (4)
O4^v^—Rb1—O4^viii^	114.1 (3)	O4^v^—P2—O4^vi^	109.1 (4)
O4^iii^—Rb1—O4^viii^	45.39 (10)	O3—P2—Rb1^xiv^	125.96 (9)
O4^vii^—Rb1—O4^viii^	93.4 (2)	O4—P2—Rb1^xiv^	54.5 (2)
O2^i^—Rb1—O4^i^	86.37 (11)	O4^v^—P2—Rb1^xiv^	54.5 (2)
O2^ii^—Rb1—O4^i^	65.15 (8)	O4^vi^—P2—Rb1^xiv^	124.2 (4)
O2^iii^—Rb1—O4^i^	85.85 (12)	O3—P2—Rb1^xv^	125.96 (9)
O2—Rb1—O4^i^	125.48 (7)	O4—P2—Rb1^xv^	54.5 (2)
O2^iv^—Rb1—O4^i^	134.74 (4)	O4^v^—P2—Rb1^xv^	124.2 (4)
O2^v^—Rb1—O4^i^	179.2 (3)	O4^vi^—P2—Rb1^xv^	54.5 (2)
O4^vi^—Rb1—O4^i^	114.1 (3)	Rb1^xiv^—P2—Rb1^xv^	89.01 (13)
O4^v^—Rb1—O4^i^	93.4 (2)	O3—P2—Rb1	125.96 (9)
O4^iii^—Rb1—O4^i^	93.4 (2)	O4—P2—Rb1	124.2 (4)
O4^vii^—Rb1—O4^i^	45.39 (10)	O4^v^—P2—Rb1	54.5 (2)
O4^viii^—Rb1—O4^i^	53.9 (2)	O4^vi^—P2—Rb1	54.5 (2)
O1^ix^—Fe1—O4^x^	113.1 (2)	Rb1^xiv^—P2—Rb1	89.01 (13)
O1^ix^—Fe1—O4^xi^	113.1 (2)	Rb1^xv^—P2—Rb1	89.01 (13)
O4^x^—Fe1—O4^xi^	105.6 (3)	P1—O1—Fe1^xviii^	180.0
O1^ix^—Fe1—O4	113.1 (2)	P1—O2—Co1	133.3 (6)
O4^x^—Fe1—O4	105.6 (3)	P1—O2—Rb1	103.9 (3)
O4^xi^—Fe1—O4	105.6 (3)	Co1—O2—Rb1	100.42 (9)
O1^ix^—Fe1—Rb1^xiv^	128.32 (5)	P1—O2—Rb1^xiv^	103.9 (3)
O4^x^—Fe1—Rb1^xiv^	52.81 (12)	Co1—O2—Rb1^xiv^	100.42 (9)
O4^xi^—Fe1—Rb1^xiv^	118.5 (2)	Rb1—O2—Rb1^xiv^	115.6 (3)
O4—Fe1—Rb1^xiv^	52.81 (12)	P2—O3—Co1^xix^	180.0
O1^ix^—Fe1—Rb1^xv^	128.32 (5)	P2—O4—Fe1	137.0 (6)
O4^x^—Fe1—Rb1^xv^	118.5 (2)	P2—O4—Rb1^xiv^	102.8 (2)
O4^xi^—Fe1—Rb1^xv^	52.81 (12)	Fe1—O4—Rb1^xiv^	100.21 (8)
O4—Fe1—Rb1^xv^	52.81 (12)	P2—O4—Rb1^xv^	102.8 (2)
Rb1^xiv^—Fe1—Rb1^xv^	85.60 (7)	Fe1—O4—Rb1^xv^	100.21 (8)
O1^ix^—Fe1—Rb1^xvi^	128.32 (5)	Rb1^xiv^—O4—Rb1^xv^	114.1 (3)

Symmetry codes: (i) −*x*+*y*+1, −*x*+2, *z*; (ii) −*y*+1, *x*−*y*+1, *z*; (iii) *x*−1, *y*, *z*; (iv) −*x*+*y*, −*x*+1, *z*; (v) −*y*+1, *x*−*y*, *z*; (vi) −*x*+*y*+1, −*x*+1, *z*; (vii) *x*, *y*+1, *z*; (viii) −*y*, *x*−*y*, *z*; (ix) *x*, *y*−1, *z*−1; (x) −*x*+*y*+2, −*x*+1, *z*; (xi) −*y*+1, *x*−*y*−1, *z*; (xii) *x*, *y*, *z*+1; (xiii) −*y*+2, *x*−*y*+1, *z*; (xiv) *x*+1, *y*, *z*; (xv) *x*, *y*−1, *z*; (xvi) *x*+1, *y*−1, *z*; (xvii) *x*+1, *y*+1, *z*; (xviii) *x*, *y*+1, *z*+1; (xix) *x*, *y*, *z*−1.
